# Oncogenic fusion proteins adopt the insulin-like growth factor signaling pathway

**DOI:** 10.1186/s12943-018-0807-z

**Published:** 2018-02-19

**Authors:** Haim Werner, Shilhav Meisel-Sharon, Ilan Bruchim

**Affiliations:** 1 Department of Human Molecular Genetics and Biochemistry, Sackler School of Medicine, Tel Aviv University, 69978 Tel Aviv, Israel; 2 Yoran Institute for Human Genome Research, Tel Aviv University, 69978 Tel Aviv, Israel; 3 Department of Obstetrics and Gynecology, Hillel Yaffe Medical Center, Hadera 38100, affiliated with the Technion Institute of Technology, Haifa, Israel

**Keywords:** Insulin-like growth factor-1 (IGF1), IGF1 receptor (IGF1R), Chimeric fusion proteins, Disrupted transcription factors, Transcription

## Abstract

The insulin-like growth factor-1 receptor (IGF1R) has been identified as a potent anti-apoptotic, pro-survival tyrosine kinase-containing receptor. Overexpression of the *IGF1R* gene constitutes a typical feature of most human cancers. Consistent with these biological roles, cells expressing high levels of IGF1R are expected *not to die*, a quintessential feature of cancer cells. Tumor specific chromosomal translocations that disrupt the architecture of transcription factors are a common theme in carcinogenesis. Increasing evidence gathered over the past fifteen years demonstrate that this type of genomic rearrangements is common not only among pediatric and hematological malignancies, as classically thought, but may also provide a molecular and cytogenetic foundation for an ever-increasing portion of adult epithelial tumors. In this review article we provide evidence that the mechanism of action of oncogenic fusion proteins associated with both pediatric and adult malignancies involves transactivation of the *IGF1R* gene, with ensuing increases in IGF1R levels and ligand-mediated receptor phosphorylation. Disrupted transcription factors *adopt* the IGF1R signaling pathway and elicit their oncogenic activities via activation of this critical regulatory network. Combined targeting of oncogenic fusion proteins along with the IGF1R may constitute a promising therapeutic approach.

## Background

The insulin-like growth factors (IGFs) constitute a family of cellular and secreted factors with important biochemical, cellular and physiological roles [1, 2]. Since their discovery in the mid-1950, the IGFs have attracted the attention of basic and clinical scientists, including developmental biologists, endocrinologists, pediatricians and oncologists [3, 4]. The IGF ligands comprise IGF1, IGF2, insulin and a number of non-classical ligands whose biological functions are still a matter of debate. IGF1 is regarded as a progression factor that is required by cells to advance through the various phases of the cell cycle [5]. The concentration of IGF1 in serum is strictly dependent on growth hormone (GH)-stimulated liver production. In addition to its classical endocrine role, a number of extra-hepatic organs, including the brain, kidney and stomach, produce IGF1 and IGF2 [6]. At the local level, IGFs display paracrine and autocrine modes of action and are able to interact with locally produced factors, including steroid hormones, extracellular matrix proteins and others. Highest IGF1 levels are measured during puberty and decrease at adult stages. IGF2 is regarded as an important growth factor during embryonic development, being its production less dependent on GH stimulation [7]. *IGF2* constitutes a typical example of an epigenetically regulated gene. *IGF2* is monoallelically expressed in somatic tissues, whereas it is usually expressed from both alleles in malignantly transformed cells [8]. Finally, *IGF2* is focally expressed early in tumorigenesis, providing critical oncogenic signals [9].

## The IGF1 receptor: A key player in cancer

IGF1 and IGF2 activate a common, ubiquitously expressed receptor, the IGF1 receptor (IGF1R), which signals mitogenic, antiapoptotic and transforming activities [10, 11]. The IGF1R is a cell-surface tyrosine kinase receptor coupled to a number of intracellular second messenger pathways, including the *ras-raf*-MAPK and PI3K signaling cascades. The IGF1R is vital for cell survival, as illustrated by the lethal phenotype of mice in which the *IGF1R* gene was disrupted by homologous recombination. During normal ontogenesis, the *IGF1R* gene is expressed at every developmental period, including the oocyte stage [12]. Late embryonic and adult phases, in which the percentage of actively proliferating cells declines, are associated with an overall reduction in IGF1R concentrations [13]. Disruption of the *IGF1R* gene resulted in small (more than 50% reduction in weight) animals that died in the immediate postnatal period from respiratory failure [14]. These animals exhibited generalized developmental abnormalities, including hypoplasia, abnormal skin formation, delayed bone development and anomalous central nervous system morphology [15].

Clinical and experimental studies provide evidence that most tumors and transformed cells display augmented cell-surface IGF1R levels (leading to enhanced IGF binding) and express high IGF1R mRNA levels compared to non-transformed cells [16–18]. In the context of cancer, the IGF1R exhibits important features that are critical for the cellular events associated with the tumorigenic process. These attributes include: (i) mitogenic and antiapoptotic capacities; (ii) crucial roles in migration, metastasis and angiogenesis; and (iii) vital role in oncogenic transformation. Increased *IGF1R* expression and ligand-mediated activation of the cell-surface IGF1R are regarded as fundamental prerequisites for acquisition of a malignant phenotype [19]. Consistent with this dogma, fibroblasts derived from *IGF1R*-null embryos do not undergo transformation when exposed to most oncogenes [20]. It is important to emphasize, however, that neither the ligand-activated nor the unligated receptor are genotoxic, i.e., IGF1R is unable, per se, to induce mutations or to cause DNA damage [21].

The above paradigm, however, is not necessarily true for *every* type of cancer. Thus, whereas IGF1R overexpression is a common feature of most pediatric tumors, often associated with recurrent chromosomal translocations (see below) and other solid tumors such as brain and renal cancers, the situation in epithelial tumors, which are more widespread in adults (e.g., breast, prostate), is more intricate and involves a tight interplay with additional cellular factors, including steroid hormones [22–26]. The vast amount of information generated in recent years by experimentalists, clinicians and epidemiologists led to the development of molecular tools aimed at targeting the IGF axis as a clinically relevant therapeutic target in oncology [27–30]. Specifically, multiple studies have evaluated more than thirty drugs targeting the IGF1R pathway, including anti-IGF1R antibodies, small molecular weight tyrosine kinase inhibitors and antibodies against IGF1 and IGF2 ligands. More than ten IGF/IGF1R inhibitors have entered clinical studies and showed sustained response in a small number of patients with select tumor types but many large clinical trials involving patients with adult tumors, including non-small cell lung, breast and pancreatic cancers failed to show clinical benefit in the overall patient population [31]. In order to be able to design more efficient targeting approaches, there is an urgent need to better understand the molecular mechanisms that govern *IGF1R* expression and action. In parallel, it is imperative to identify biomarkers that can predict responsiveness to IGF1R-directed therapies [29, 32, 33].

## Transcriptional regulation of *IGF1R* gene expression

The net level of expression of the *IGF1R* gene in both normal and malignant cells is determined, to a large extent, at the transcriptional level [11, 19]. Basal transcription rate is dependent on a number of stimulatory nuclear proteins, including zinc-finger specificity protein-1 (Sp1) [34, 35], E2F1 [36], high mobility group protein A1 (HMGA1) [37], and others. The rate of *IGF1R* transcription is also dependent on the presence of inhibitory transcriptional regulators, including p53 [38–41], breast and ovarian cancer gene-1 (BRCA1) [22, 42–44], Wilm’s tumor protein (WT1) [45], etc. It has been postulated that the etiology of tumors associated with *loss-of-function* mutation of tumor suppressor genes is linked to the inability of mutated (inactive) tumor suppressor proteins to repress their downstream targets, including the *IGF1R* gene [19]. *Gain-of-function* mutations of oncogenes, on the other hand, lead to enhanced transactivation of the *IGF1R* promoter. Increased cell-surface IGF1R concentrations are usually correlated with augmented receptor activation by circulating and locally produced IGF1/IGF2, with ensuing mitogenic responses [46]. Interplay between stimulatory and inhibitory transcriptional regulators as well as interactions with additional, tissue-specific factors, may impinge upon the proliferative status of the cell. The participation of oncogenic transcription factors in regulation of *IGF1R* gene expression is described below.

## Disrupted transcription factors: A common theme in oncology

Tumor specific chromosomal translocations that disrupt the genomic organization of transcription factors are a common theme in oncogenesis [47, 48]. As a result of these rearrangements, chimeric proteins are generated that are composed of modules derived from unrelated genes. Most prototypical cases of aberrant chimeras have been described in the context of pediatric and hematological malignancies [49]. One of the classical examples is the oncogenic BCR-ABL tyrosine kinase fusion, the product of the Philadelphia chromosome, which results from a recurrent translocation between chromosomes 9 and 22 and constitutes the hallmark of chronic myelogenous leukemia (CML) [50, 51]. A second example of a well-characterized rearrangement is the translocation of the *c-myc* gene in Burkitt’s lymphoma. In this pathology, the *c-myc* proto-oncogene is juxtaposed to an immunoglobulin gene by chromosomal fusion, thereby activating the oncogene. Extensive research on the biology of these (and other) aberrant transcription factors over the past decades had a huge impact on our current understanding of the mechanistic events associated with chromosomal translocations and their roles in cancer etiology [52]. Furthermore, the recent introduction of genomic and proteomic platforms is allowing the identification of genes and signaling pathways that are involved in the pathological chain of biochemical events associated with disrupted transcription factors. Table 1 presents a summary of common fusion proteins and their interactions with the IGF1 system.

**Table 1:** Examples of oncogenic fusion proteins and their interactions with the IGF1R

Fusion protein	Chromosomes involved	Type of tumor	Interaction with IGF1R
BCR-ABL	t(9;22)	Chronic myelogenous leukemia	IGF1R regulates the cell fate determination of *BCR-ABL*+ leukemia cells
EWSR1-FLI1	t(22;11)	Ewing sarcoma	An intact IGF1R is required for the oncogenic action of EWSR1-FLI1
TMPRSS2-ERG	t(21;22)	Prostate cancer	Overexpression of ERG leads to transactivation of the *IGF1R* gene
MYB-NFIB	t(6;9)	Adenoid cystic carcinoma (salivary gland, others)	The fusion protein is regulated via IGF1R-stimulated AKT activation
ETV6-NTRK3	t(12;15)	Congenital fibrosarcoma, others	ETV6-NTRK3 forms a tripartite complex with phosphorylated IRS1 and IGF1R. This complex is critical for the oncogenic action of the chimera
EWSR1-WT1	t(11;22)	Desmoplastic small round cell tumor	The chimera transactivates the *IGF1R* gene

## Pediatric and adolescent tumors

As referred to above, several pediatric and adolescent tumors are characterized by recurrent chromosomal translocations, frequently resulting in the fusion of unrelated genes, most commonly transcription factors and nucleic acid-binding proteins. An example of this growing family of dislocated transcription factors is the t(11; 22) chromosomal translocation found in nearly 90% of Ewing’s sarcomas (EWS) and primitive neuroectodermal tumor of childhood (PNET). This specific translocation results in the fusion of the 5′ end of the ubiquitously expressed *EWSR1* gene on chromosome 22 to the 3′ end of the *FLI1* gene on chromosome 11 [53–55]. The *EWSR1* gene is a member of the *TET* gene family [56], a class of RNA-binding proteins of unknown physiological function [57]. The *EWSR1* gene is involved in a number of translocation events that are hallmarks of a variety of specific cancers [58]. The chimeric gene product EWSR1-FLI1 contains the transcriptional domain of EWSR1, which is usually involved in protein-protein interactions, and the DNA-binding domain of FLI1.

Desmoplastic small round cell tumor (DSRCT), an aggressive primitive tumor of children and young adults, particularly males [59], is also characterized by a recurrent translocation involving the *EWSR1* gene. The distinctive hallmark of DSRCT is the t(11;22)(p13;q12) translocation [60] that fuses the N-terminal transcriptional activation domain of *EWSR1* to the C-terminal DNA-binding domain of Wilm’s tumor-1 (*WT1)* [61–63]. WT1 is a zinc finger-containing tumor suppressor shown to be involved in the etiology of Wilm’s tumor or nephroblastoma, a pediatric kidney malignancy [64]. Consistent with the postulate that the EWSR1-WT1 fusion protein is capable of modulating transcription of target genes containing WT1 binding motifs, we have shown that the chimera can recognize and transactivate the *IGF1R* promoter in transient transfection assays (see below) [65, 66].

## Sarcomas and chondrosarcomas

Fusion of the *EWSR1* and *ATF-1* genes is characteristic of soft tissue clear cell sarcoma or malignant melanoma of soft parts [67]. A recurrent t(9; 22)(q22; q12) chromosome translocation has been described in extraskeletal myxoid chondrosarcoma. As a result of this rearrangement, the *EWSR1* gene fuses to an orphan nuclear receptor gene, initially named TEC [68]. Although these fusions exhibit heterogeneity with respect to the chromosomal breakpoints, a common feature is the fusion of the N-terminal transcription activation domain of EWSR1 to either the full-length sequence or the C-terminal DNA-binding domain of any of a number of transcription factors [69, 70]. Antisense or antibody inhibition of the chimeric EWSR1-FLI1 and EWSR1-ATF1 molecules reduces the tumorigenicity and viability of Ewing’s and clear cell carcinoma cells, respectively, implicating the altered transcriptional activity of these chimeric proteins in tumor development.

## ETV6-NTRK3 fusion protein

The ETV6-NTRK3 chimera was discovered by breakpoint analysis of the t(12;15)(p13;q25) translocation characteristic of congenital fibrosarcoma, a pediatric soft tissue malignancy. The fusion protein includes the sterile alpha motif oligomerization domain of the ETV6 transcription factor linked to the tyrosine kinase domain of the neurotrophin-3 receptor NTRK3 [71]. This aberrant protein has been shown to be linked to a number of signaling cascades, including the *ras-raf*-MAPK and PI3K networks. Following its initial identification in congenital fibrosarcoma, as mentioned above, the ETV6-NTRK3 chimera was also detected in tumors derived from different cell lineages, including secretory breast carcinoma [72] and inflammatory myofibroblastic tumors [73].

The oncogenic activity of ETV6-NTRK3 is tightly dependent on IGF1R action and, furthermore, insulin receptor substrate-1 (IRS1) is constitutively phosphorylated in chimera-transformed cells [74]. Of interest, ETV6-NTRK3 forms a tripartite complex with phosphorylated IRS1 and IGF1R that seems to be critical for the oncogenic action of the chimera. ETV6-NTRK3 colocalizes with IGF1R at the plasma membrane. In agreement with this physical and functional interplay, targeting of the IGF1R leads to blockade of ETV6-NTRK3-mediated breast epithelial transformation [75]. The interactions between additional chimeric proteins and the IGF1R are described below.

## Adult cancers

Cytogenetic profiling of all major cancers led to the widespread dogma that recurrent translocations underlie the development of sarcomas and hematological malignancies whereas, on the other hand, adult epithelial tumors are rarely correlated with this type of rearrangements [76]. In recent years, however, evidence accumulated demonstrating that this type of genetic events may also explain some adult tumors. Using a bioinformatic approach aimed at discovering candidate oncogenic chromosomal aberrations on the basis of outlier gene expression, Tomlins et al. [77] reported the identification of recurrent gene fusions of the 5′ untranslated region of the *TMPRSS2* gene to the *ERG* or *ETV1* genes in prostate cancer. The *TMPRSS2* gene is located on chromosome 21 and is highly expressed in prostate epithelium [78]. The gene encodes a 492-amino acid serine protease with five distinct domains, including a transmembrane region [79]. While the normal function of TMPRSS2 is unknown, *TMPRSS2* has been identified as an androgen-sensitive gene. Fusion of this gene to members of the ETS family of transcription factors, in particular oncogenes *ERG* or *ETV1*, leads to over-expression of these transforming agents in a significant portion of prostate cancers, but not benign prostate tissue, in an androgen-dependent manner [80].

**Fig. 1:**
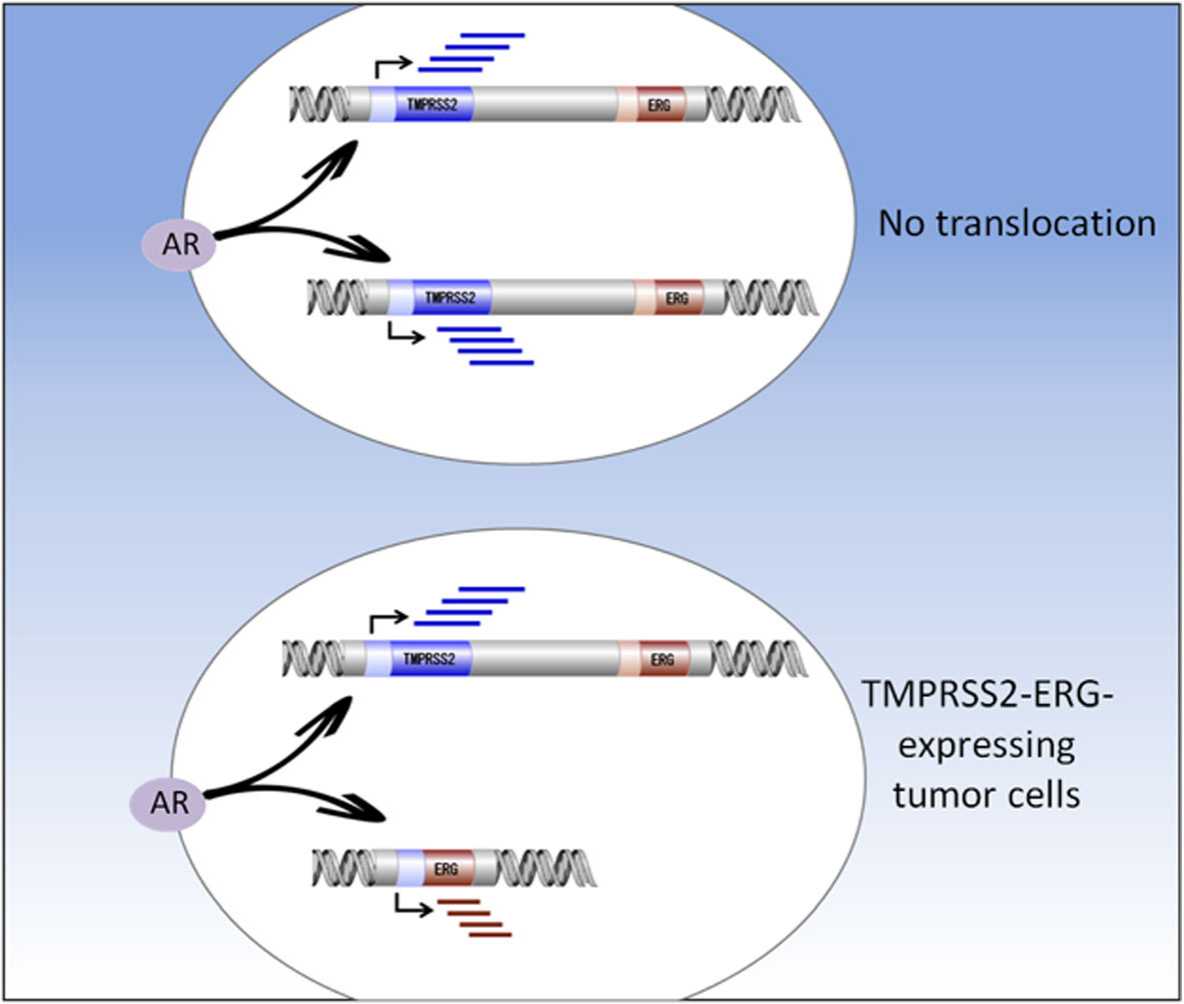
Juxtaposition of *TMPRSS2* to *ERG* genes in prostate cancer leads to oncogene ERG overexpression. Gene fusion of the 5′ untranslated region of the *TMPRSS2* gene to the *ERG* or *ETV1* genes in prostate cancer is recognized as one of the most common rearrangements in oncology. In benign cells not expressing the translocation product, androgens (acting via the androgen receptor, AR) stimulate *TMPRSS2* gene transcription. *ERG* does not seem to undergo enhanced transcription in non-malignant cells. On the other hand, in prostate epithelial cells expressing the TMPRSS2-ERG fusion protein, AR promotes transcription of the ERG oncogene by virtue of its fusion to the androgen sensitive *TMPRSS2* promoter

The identification of TMPRSS2-ERG as an important player in prostate cancer etiology had a major impact in basic and translational oncology [76]. Recurrent chromosomal translocations leading to pathologic production of disrupted transcription factors are now recognized as a relatively common event in adult epithelial tumors. For the most part, aberrant transcription factors exhibit *gain-of-function* (usually oncogenic) activities that abrogate the intrinsic biological role of each of the parental genes. In the specific case of TMPRSS2-ERG, the androgen responsiveness of the *TMPRSS2* promoter is responsible for the steroid-dependent expression of oncogene ERG in prostate gland epithelium, a key event in prostate carcinogenesis. In the clinical context, reports identified a statistically significant association between TMPRSS2-ERG fusion and prostate cancer specific death [81]. Hence, data is consistent with chimera-expressing tumors having a more aggressive phenotype (Fig. 1). Of importance, the TMPRSS2-ERG fusion protein has been shown to impinge upon several oncogenic pathways relevant to prostate cancer etiology. A list of recently identified TMPRSS2-ERG downstream targets and signaling pathways is presented in Table 2.

**Table 2:** TMPRSS2-ERG target genes and signaling pathways

Targeted pathway	Biological effect	Reference
TGF-β1	Increased migration and invasiveness; reduced proliferation and accumulation in G1 phase; epithelial to mesenchymal transition.	[98]
RUNX2	Destabilization of bone metabolism; effect on bone metastasis.	[99]
Frizzled 4/WNT	Changes in WNT signaling; effect on cell adhesion.	[100]
NOTCH	Effects on cell growth, survival and apoptosis.	[101]

A novel, cancer-specific gene fusion between *BCAM*, a membrane adhesion molecule, and *AKT2*, a member of the PI3K signaling pathway, was recently described in high-grade serous ovarian cancer cases [82]. This rearrangement leads to translation of a fused BCAM-AKT2 protein in patient’s tumors. The chimera exhibits a membrane location and is constitutively phosphorylated. BCAM-AKT2 functions as a kinase in malignant cells and, unlike endogenous AKT2 whose activity is governed by external stimuli, the chimeric protein is active in a steady fashion. This oncogenic fusion is present in 7% of high-grade serous cancers and constitutes the hallmark of a novel subclass of ovarian cancers. In addition, the chimera may, possibly, provide a therapeutically relevant target. The interactions between the IGF1 signaling pathways and disrupted oncogenic proteins are discussed below.

## Transcriptional regulation of *IGF1R* by oncogenic fusion proteins

**Fig. 2:**
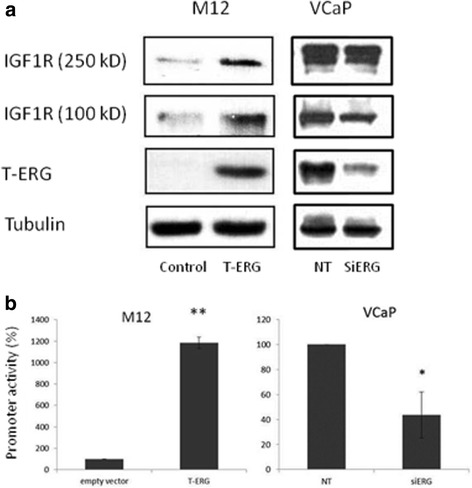
Regulation of *IGF1R* gene expression by aberrant transcription factor TMPRSS2-ERG in prostate cancer cells. **a** M12 cells (lacking the fusion protein) were infected with an ERG-encoding viral vector. Cells were lysed, electrophoresed through SDS-PAGE, followed by transfer and incubation with an IGF1R β subunit antibody. ERG expression leads to enhancement of both the mature (100-kDa) and precursor (250-kDa) forms of IGF1R. VCaP cells (expressing TMPRSS2-ERG) were transfected with a siRNA directed against the fusion protein (siERG), or control non-targeting (NT) siRNA. Cells were harvested after 96 h and levels of T-ERG and IGF1R were measured by Western blots. Data indicate that ERG silencing led to a reduction in mature IGF1R levels. **b** M12 cells were cotransfected with an IGF1R promoter-luciferase reporter, along with an ERG expression vector (or empty vector), and VCaP cells were cotransfected with the IGF1R luciferase reporter, along with siERG or NT siRNA. Luciferase activity was measured after 48 h and normalized to β-galactosidase values. Results indicate that ERG stimulated *IGF1R* gene transcription [83]

**Fig. 3:**
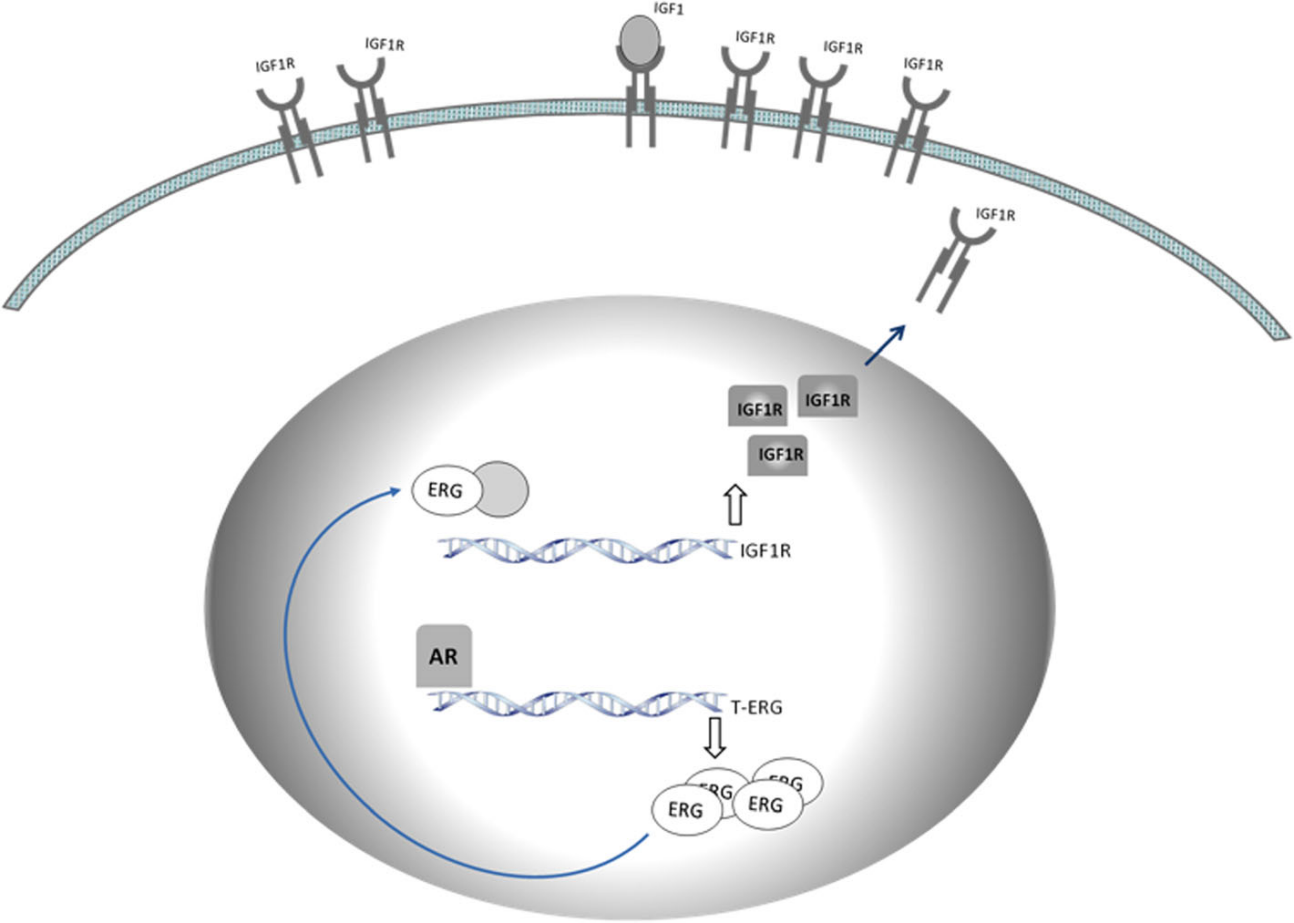
Model for TMPRSS2-ERG regulation of the *IGF1R* gene. Schematic representation of the activation of the *IGF1R* gene by oncogenic ERG. In prostate cancer cells expressing the chimera, androgens, acting via the androgen receptor (AR), promote transcription of the ERG oncogene. Androgen-stimulated ERG expression results from the fusion of *ERG* to the androgen sensitive *TMPRSS2* promoter. Subsequently, ERG accumulates and transactivates the *IGF1R* promoter either directly or through complex protein-protein interactions with a series of DNA-binding proteins. Enhanced *IGF1R* gene expression leads to high levels of expression of the receptor at the cell surface, with ensuing activation by locally produced or circulating IGF1 and/or IGF2. IGF1R overexpression is a typical feature of most cancers and transformed cell lines

As mentioned above, IGF1R exhibits anti-apoptotic, pro-survival activities, and is regarded as a key player in cancer. It is therefore relevant to question whether the mechanism of action of oncogenic fusion proteins involves transactivation of the *IGF1R* gene. The rationale for this postulate is the fact that *IGF1R* is frequently overexpressed in tumors associated with chromosomal translocations. To examine the hypothesis that the prostate-specific TMPRSS2-ERG fusion is able to transactivate the *IGF1R* gene, M12 prostate cancer cells were infected with an ERG-encoding retroviral vector, followed by IGF1R expression measurements. Western blots and qRT-PCR indicate that ERG expression led to a marked increase in IGF1R protein and mRNA levels compared to uninfected cells (Fig. 2a). On the other hand, ERG silencing in VCaP cells (expressing an endogenous TMPRSS2-ERG) reduced IGF1R levels. Transient co-transfection assays using an *IGF1R* promoter-luciferase reporter plasmid along with an ERG vector led to a major increase in *IGF1R* promoter activity, indicating that the effect of the oncogene was mediated at the transcriptional level (Fig. 2b). Data is consistent with the notion that the mechanism of action of the fusion protein involves transactivation of the *IGF1R* gene, with ensuing increases in IGF1R levels and ligand-mediated receptor phosphorylation [83] (Fig. 3). Similar results showing a positive correlation between fusion protein and IGF1R expression in prostate cancer lines and clinical samples were reported by Mancarella et al. [84].

An additional chimeric transcription factor whose mechanism of action involves transactivation of the *IGF1R* gene is the EWSR1-WT1 oncogene, the typical hallmark of DSCRT. As alluded to above, fusion of EWSR1 to WT1 in this pediatric malignancy abrogates the tumor suppressor role of WT1 as well as the RNA-binding capacity of EWSR1, and generates an oncogenic molecule capable of binding and transactivating WT1 target genes, including the *IGF1R* promoter [65, 85]. Hence, whereas wild type WT1 suppressed *IGF1R* transcription, the pathogenic EWSR1-WT1 fusion molecule leads to enhanced *IGF1R* transcription, with ensuing activation of cell-surface receptors by circulating and locally produced IGF1 and/or IGF2.

A further malignancy characterized by a recurrent chromosomal translocation is alveolar rhabdomyosarcoma, a pediatric soft tissue tumor. The t(2;13)(q35;q14) translocation characteristic of the disease juxtaposes the 5’-DNA binding domain-encoding sequences of the *PAX3* gene with the 3′ sequences of the *FKHR* gene to generate a *PAX3-FKHR* chimera [86–89]. PAX3 is a developmentally regulated nuclear protein that is expressed in muscle progenitor cells, while FKHR is a member of the forkhead family of transcription factors. The PAX3-FKHR chimera has been shown to function as an aberrant transcription factor. The involvement of this disrupted protein in tumorigenic cell growth seems to result from the alteration of DNA binding and transactivation activity and/or deregulation of *PAX3* expression [90]. Transfection of sarcoma-derived cell lines with expression vectors encoding PAX3-FKHR resulted in transactivation of a co-transfected *IGF1R* promoter construct, whereas PAX3 exhibited a reduced potency in comparison to the chimera [91]. These results provide a mechanistic basis for the increased IGF1R levels observed in rhabdomyosarcoma. The importance of IGF1R action in progression of these tumors is underscored by studies showing that a monoclonal antibody against IGF1R inhibited the growth of rhabdomyosarcoma xenografts in mice [92].

## An intact IGF1R is required for oncogenic action of chimeric proteins

While studies described above support the idea that the mechanism of action of oncogenic fusion proteins involves transactivation of *IGF1R*, it has been postulated that the opposite scenario, i.e. that the presence of an intact IGF1R is critical for the oncogenic action of the chimeras, is also biologically plausible. To evaluate the hypothesis that the transforming activity of EWSR1-FLI1 requires the presence of IGF1R, W and R- mouse fibroblasts (containing or lacking, respectively, IGF1R) were transfected with a chimera-expressing vector, after which anchorage-independent growth was assessed [93]. Results of soft agar assays indicated that W, but not R-, cells grew in an anchorage-independent fashion upon chimera transfection. IGF1R presence is, therefore, a critical prerequisite for EWSR1-FLI1 transformation.

Further support for a crucial role of IGF1R in leukemia was provided by studies showing that IGF1R regulates the cell fate determination of *BCR-ABL*+ leukemia cells and supports the self-renewal of CML cells [94]. IGF1R expression was shown to be significantly higher in CML than in acute lymphoblastic leukemia (ALL). Lack of IGF1R resulted in decreased self-renewal of the *BCR-ABL*+ CML cells. Therefore, IGF1R directs *BCR-ABL*+ leukemia cells toward the myeloid fate. Given the fact that IGF1R is dispensable for the activity of hematopoietic cells but regulates *BCR-ABL* leukemia cell fate and supports self-renewal of CML cells, targeting IGF1R has been suggested to constitute an ideal anti-leukemia approach.

Finally, the MYB-NFIB gene fusion has been identified as the characteristic hallmark of adenoid cystic carcinoma (ACC), an aggressive type of cancer that most often occurs in the salivary gland. Recent studies performed on cultured cells or tumors from ACC patients provided evidence that MYB-NFIB fusion drives proliferation and is crucial for spherogenesis of these cells. Furthermore, the fusion was shown to be regulated through AKT-dependent signaling induced by IGF1R overexpression. IGF1R inhibition, on the other hand, led to downregulation of MYB-NFIB action. Given the potential role of the chimera as a therapeutic target, its interaction with the IGF1R is of major translational relevance [95].

## Chimeric transcription factors adopt the IGF1 signaling pathways

Activation of the IGF1R signaling pathway in a ‘*cascade’* fashion (i.e., phosphorylation of the receptor tyrosine kinase domain with ensuing activation of downstream signaling molecules) is regarded as a fundamental requirement in transformation. As mentioned above, both viral and cellular oncogenes require an intact IGF1R signaling network in order to elicit their transforming activities. This phenomenon is commonly regarded as ‘*adoption*’ of the IGF1R pathway by oncogenic agents [19].

In mechanistic terms, the modes of action of oncogenic fusion proteins depend on their ability to phosphorylate the IGF1R and downstream cytoplasmic elements. In the specific case of the prostate cancer-specific TMPRSS2-ERG aberrant transcription factor, we have demonstrated that the chimera was able to phosphorylate the IGF1R tyrosine kinase domain and downstream target Akt. ERG silencing in TMPRSS2-ERG-containing VCaP prostate cells led to a reduction in IGF1R expression. Decreased IGF1R levels were associated with a reduction in IGF1R activation as well as diminished Akt phosphorylation [83, 84]. In the patho-physiological context of prostate cancer, TMPRSS2-ERG fusion protein is presumably functioning in the presence of the wild type, untranslocated ERG protein. The interactions between the translocation product and the full-length, untranslocated, ERG, are still unclear.

Finally, activation of IGF1R target elements by oncogenic chimeras, i.e., *adoption* of the IGF1R signaling pathway, seems to be a universal mechanism of action of oncogenes. Thus, transformation by pp60^src^, the protein encoded by the *src* oncogene of Rous sarcoma virus, results in the constitutive phosphorylation of the IGF1R β subunit [96]. It has been estimated that ~ 10–50% of the receptors are phosphorylated in the unstimulated *src*-transformed cell while addition of IGF1 synergistically increased the extent of phosphorylation. In a similar fashion, the hepatitis B virus X protein was shown to enhance IGF1R mRNA levels in hepatocellular cancer cell lines. These results suggest that the oncogenic role of hepatitis B virus X is mediated via transactivation of the *IGF1R* gene [97]. In summary, oncogenes alter growth regulation by rendering the cells constitutively subject to a mitogenic signal.

## Conclusions

The IGF1R has been identified as a potent anti-apoptotic, pro-survival tyrosine kinase-containing receptor. Cells expressing high levels of cell-surface IGF1R are expected *to survive*, a hallmark of cancer cells. Overexpression of the *IGF1R* gene constitutes a typical feature of most human cancers. *IGF1R* gene expression is determined, to a significant extent, at the transcriptional level. Evidence has been presented showing that the *IGF1R* promoter constitutes a target to a number of aberrant transcription factors that result from recurrent, cancer-specific chromosomal translocations. The classical dogma that disrupted transcription factors are mainly associated with pediatric and hematological malignancies has been challenged in recent years by landmark studies showing that the etiology of a number of adult epithelial cancers is also linked to oncogenic chimeric proteins. Oncogenes *adopt* the IGF1R signaling pathway and elicit their transforming activities via activation of this critical network. Combined targeting of oncogenic fusion proteins along with the IGF1R may constitute a promising therapeutic approach in oncology and may overcome the difficulties seen with IGF1R-directed monotherapy.
